# Comparison of Diagnostic Efficacy of [^68^Ga]Ga-FAPI-04 and [^18^F]FDG PET/CT for Staging and Restaging of Gastric Cancer

**DOI:** 10.3389/fonc.2022.925100

**Published:** 2022-07-01

**Authors:** Shumao Zhang, Wei Wang, Tingting Xu, Haoyuan Ding, Yi Li, Huipan Liu, Yinxue Huang, Lin Liu, Tao Du, Yan Zhao, Yue Chen, Lin Qiu

**Affiliations:** ^1^ Department of Nuclear Medicine, The Affiliated Hospital of Southwest Medical University, Luzhou, China; ^2^ Nuclear Medicine and Molecular Imaging Key Laboratory of Sichuan Province, Luzhou, China; ^3^ Institute of Nuclear Medicine, Southwest Medical University, Luzhou, China; ^4^ Department of Geriatric Psychiatry, The Fourth People’s Hospital of Chengdu, Chengdu, China; ^5^ Department of Geriatric Psychiatry, The Clinical Hospital of Chengdu Brain Science Institute, Ministry of Education (MOE) Key Lab for Neuroinformation, University of Electronic Science and Technology of China, Chengdu, China

**Keywords:** [^68^Ga]Ga-FAPI-04, [^18^F]-FDG, gastric cancer, tumor staging, PET/CT

## Abstract

**Purpose:**

This study aimed to compare the potential diagnostic efficacy of gallium68-fibroblast-activation protein inhibitor ([^68^Ga]Ga-FAPI-04) and fluorine18-fluorodeoxyglucose ([^18^F]-FDG) positron emission tomography-computed tomography (PET/CT) for primary tumors, lymph nodes, and distant metastatic lesions of gastric cancer (GC), and to explore the effects of [^68^Ga]Ga-FAPI-04 and [^18^F]-FDG on tumor staging and restaging in GC.

**Methods:**

This single-center retrospective study (NCT2100044131) was conducted at the Affiliated Hospital of the Southwest Medical University between June 2020 and December 2021. Images of patients with GC who were pathologically confirmed and underwent contemporaneous [^18^F]-FDG and [^68^Ga]Ga-FAPI-04 PET/CT within 1 week were analyzed. The diagnostic efficacy of [^68^Ga]Ga-FAPI-04 PET/CT and [^18^F]-FDG PET/CT for TNM staging of GC was compared using McNemar test. The maximum standard uptake value (SUVmax) of each lesion in the two imaging types was compared using the Mann-Whitney U test.

**Results:**

In total, 25 patients with GC (mean age, 56 ± 12 years) were evaluated. [^68^Ga]Ga-FAPI-04 PET/CT exhibited higher sensitivity compared to [^18^F]-FDG PET/CT for detecting primary tumors (18/19 [94.74%] vs. 13/19 [68.42%], χ2 = 6.866, P < 0.01), lymph node metastasis (75/77 [97.40%] vs. 32/77 [41.56%], χ2 = 2.888, P =0.089), and distant metastases (275/283 [97.17%] vs. 122/283 [43.11%], χ2 = 11.858, P < 0.01). [^68^Ga]Ga-FAPI-04 accumulation was significantly higher than that of [^18^F]FDG in tumors (median SUVmax, 10.28 vs 3.20; U=59.00, P < 0.01), lymph node metastasis metastases (median SUVmax, 9.20 vs 3.15; U=53.50, P < 0.01), and distant metastases (median SUVmax, 8.00 vs 4.20; U=200.00, P < 0.01). Compared to [^18^F]-FDG PET/CT, [^68^Ga]Ga-FAPI-04 PET/CT resulted in new oncological findings in 14/25 patients and corrected tumor staging or restaging in 7/25 patients.

**Conclusion:**

Our preliminary results regarding the impact of [^68^Ga]Ga-FAPI-04 PET/CT on tumor staging highlight the potential of this approach for increasing the accuracy of GC diagnosis, which may facilitate treatment decision-making.

## Introduction

Gastric cancer (GC) is the fifth most common cancer and third most common cause of cancer-related deaths worldwide (1). Adenocarcinoma accounts for more than 95% of gastric malignancies (2). Curative therapy for GC relies predominantly on complete tumor resection. Accordingly, accurate imaging-based staging and restaging of GC are key to effective treatment, which will provide the greatest benefit to patients.

Fluorine18-fluorodeoxyglucose ([^18^F]-FDG)-PET/CT is currently the most frequently used imaging modality for initial tumor staging, evaluation of treatment response, and detection of recurrence in most oncological malignancies. However, [^18^F]-FDG may not be an ideal imaging agent for GC, as several limitations have been noted. Mucinous, signet ring cell, and poorly differentiated adenocarcinomas demonstrate less [^18^F]-FDG uptake compared to other histological types of gastric adenocarcinoma (3). Further, the sensitivity of [^18^F]-FDG PET for detecting lymph node metastasis of GC is low, which is associated with the histological type of primary tumor (4). The high physiological uptake background of the normal gastric wall also reduces the sensitivity of PET for N staging. Crucially, [^18^F]-FDG PET/CT often demonstrates low detection efficiency for distant metastasis (5, 6).

Fibroblast-activating protein (FAP) was recently identified to be highly expressed in cancer-associated fibroblasts (CAFs) and is closely related to cancer cell proliferation, tumor immunity, angiogenesis, extracellular matrix remodeling, and metastasis. FAP has low expression levels in normal tissues and organs, which makes it a good molecular target for tumor diagnosis and treatment (7–9). This imaging agent has good pharmacokinetics and biological chemical distribution, high sensitivity, very clear tumor outline, and a high tumor background ratio in common solid tumors (8, 10, 11). Studies have reported that GC can be detected using [^68^Ga] Ga-FAPI-04 PET/CT (5, 6, 8, 10, 12, 13). The main purpose of this study was to compare the potential diagnostic efficacy between [^68^Ga]Ga-FAPI-04 PET/CT and [^18^F]-FDG PET/CT for detecting primary tumors, lymph nodes, and distant metastases in patients with GC.

## Material and Methods

### Patients

All procedures performed in this study involving human participants were in compliance with the ethical standards of the Clinical Research Ethics Committee of the Affiliated Hospital of Southwest Medical University (Medical Research Ethics Committee: 2020035) and China Clinical Trials Registry (Clinical Trials Registry No.ChiCTR2100044131). Written informed consent was obtained from all patients. A retrospective analysis was performed on patients with GC who underwent whole-body PET/CT at the Affiliated Hospital of Southwest Medical University between June 2020 and December 2021.

Inclusion criteria were as follows: a. patients who underwent [^18^F]-FDG PET/CT and [^68^Ga]Ga-FAPI-04 PET/CT for tumor staging and restaging to determine the most appropriate treatment strategy. b. GC diagnosed by pathology or follow-up. Exclusion criteria were as follows: a. pregnant patients or patients under 18 years of age. b. participants and their parents or legal representatives were unable or unwilling to provide written informed consent. A final total of 25 patients were included.

### Tracer Synthesis

[^18^F]-FDG was manufactured according to the standard method used in our laboratory using the coincidence [^18^F]-FDG synthesis module (FDG-N, PET Science & Technology). DOTA-FAPI-04 was purchased from MedChemExpress, LLC. [^68^Ga]Ga-FAPI-04 was prepared according to a previously described protocol (14). Radioactive high-performance liquid chromatography demonstrated that the radiochemical purity of [^68^Ga]Ga-FAPI-04 was > 98%. The final products, [^18^F]-FDG and [^68^Ga]Ga-FAPI-04, were sterile and pyrogen-free.

### Imaging Protocol

The patients fasted for at least 6 h before the [^18^F]-FDG PET/CT scan. The blood glucose level of all patients was < 7.0 mmol/L (the blood glucose of patients with diabetes was < 11.1 mmol/L). Patients who underwent [^68^Ga]Ga-FAPI-04 PET/CT examinations did not require special preparation. The intravenous doses of [^18^F]-FDG and [^68^Ga]Ga-FAPI-04 were weight-adjusted (3.7 and 1.85 MBq/kg, respectively). Patients were required to drink 1,000 mL of water to fill their stomach before examination and to urinate before the PET/CT scan. 60 minutes after intravenous injection (15), PET/CT examination (uMI780, United Imaging Healthcare) was performed from the head (separate head scans for patients with suspected brain metastasis) to the middle thigh. CT scanning was performed with the following parameters: tube voltage, 120 kV; current, 120 mA; layer thickness, 3.00 mm. The scans were reconstructed using a matrix size of 128×128 pixels. Data were uploaded to a post-processing workstation (version R002, uWS-MI, United Imaging Healthcare) for processing. All PET images required iterative reconstructions.

### Image Analysis

[^18^F]-FDG and [^68^Ga]Ga-FAPI-04 PET/CT images were evaluated by two experienced nuclear medicine doctors. Any differences in opinion were resolved through consultation. On PET images, a lesion was considered positive if the focal area of [^18^F]-FDG or [^68^Ga]Ga-FAPI-04 uptake was visually higher than the background (brain: normal grey matter; other parts except the brain: mediastinal blood pool). The location, number, and SUVmax value of positive lesions were recorded. We evaluated primary tumor, lymph node metastasis, and distant metastasis of GC. Based on the guidelines of the American Joint Committee on Cancer (AJCC), T and N pathological staging was performed based on the results of postoperative pathological examination. The lymph nodes were divided into three regions: those around the gastric cavity, perivascular, and perivisceral. Involvement of the retropancreatic, mesenteric root, middle colic, para-aortic, peripancreatic, infradiaphragmatic, paraesophageal, lower thoracic, and other distant lymph nodes was considered distant metastasis (M1). Ovarian metastases were classified separately.Liver lesions with uptake higher than that of the surrounding liver parenchyma were identified as positive. The lesion number and SUVmax of the lesion with the highest pathological tracer accumulation were recorded for each lymph node region or distant metastasis site for both [^18^F]-FDG and [^68^Ga]Ga-FAPI-04 PET/CT. The upper limit of the number of regional lymph nodes or distant metastases was set at 20, and numbers > 20 were capped and recorded as 20.

### Reference Standards

All gastroscopic biopsies, surgical resection, pathological biopsies of local tissues, and imaging follow-up were considered reference standards. Due to technical and ethical issues, histopathological confirmation was not possible for all lesions especially for nodal and distant metastasis, thus the results of morphologic imaging (US, contrast-enhanced CT, MRI, or EUS), and/or follow-up imaging results were also used as the reference standard. The follow-up period was at least 3 months. Follow-up imaging studies that were considered validation of the malignant nature of lesions were either progression of metastatic disease or response to anti-cancer treatment (chemo, radio, targeted therapy, and/or immunotherapy) in terms of reduction in size and/or number of lesions. Disease progression was defined as the increase in lesion size and/or the number of metastatic lesions.

### Statistical Analysis

Statistical analysis was conducted using IBM SPSS 26.0 software (SPSS Inc). Continuous variables were evaluated for normal distribution with Shapiro-Wilk test. Categorical variables were expressed as frequencies and percentages. Continuous variables were expressed as means ± standard deviations (SDs). The diagnostic efficacy of [^68^Ga]Ga-FAPI-04 PET/CT and [^18^F]-FDG PET/CT for TNM staging of GC was compared using McNemar test. Differences in SUVmax in primary tumor, lymph node metastasis, and distant metastasis were compared between [^68^Ga]Ga-FAPI-04 and [^18^F]-FDG PET/CT using the Mann-Whitney U test. Differences were considered statistically significant at P < 0.01.

## Results

### Patient Characteristics

In total, 25 patients (13 female, age: 59.4 ± 4.9 years, range: 35-79 and 12 male, age: 51.8 ± 12.5, range: 35-70) were included in this study. The mean age was 56 ± 12 years (range: 35-79 years). Two patients had primary recurrence with distant metastasis, and two patients had distant metastasis but no primary recurrence. There were no primary recurrences or metastases in four patients. [^68^Ga]Ga-FAPI-04 and [^18^F]-FDG PET/CT was performed for restaging due to suspected progressive disease, while the 17 patients with new diagnoses underwent PET imaging for primary staging. Based on pathological type, we identified 16 cases of poorly differentiated adenocarcinoma, (including 4 cases of signet ring cell carcinoma, 1 case of signet ring cell carcinoma and mucinous adenocarcinoma), 2 cases of moderately differentiated adenocarcinoma, 2 cases of well-differentiated adenocarcinoma, and 5 cases of adenocarcinoma with unknown differentiation. Patient characteristics are presented in **Table 1**.

**Table 1:** Patient characteristics.

Patients	25
Age (y)	
Mean	56 ± 12
Interquartile range	35-79
Sex	
Female	13 (52%)
Male	12 (48%)
Histologic findings	
Well-differentiated adenocarcinoma	2 (8%)
Moderately differentiated adenocarcinoma	2 (8%)
Poorly differentiated adenocarcinoma	11 (44%)
Poorly differentiated adenocarcinoma with partial signet ring cell carcinoma	4 (16%)
Poorly differentiated adenocarcinoma with partial mucinous adenocarcinoma and signet ring cell carcinoma	1 (4%)
Unknown differentiated adenocarcinoma	5 (20%)
Indication for PET	
Initial assessment (staging)	17(68%)
Recurrence detection (restaging)	8 (32%)
Patient status	
Treatment-naive	17 (68%)
Resection surgery	1 (4%)
Chemotherapy after surgery	4 (16%)
Targeted therapy and chemotherapy after surgery	3 (12%)
Reference standards	
Gastroscopic biopsies	6 (24%)
Surgical resection	9 (36%)
Pathological biopsies and imaging follow-up	3 (12%)
Imaging follow-up	7 (28%)

### Safety

None of the patients presented with any drug-related side effects during or after [^18^F]-FDG and [^68^Ga]Ga-FAPI-04 PET/CT. PET imaging was well tolerated by all patients. None of the patients reported abnormal symptoms.

### Diagnostic Efficiency of [^68^Ga]Ga-FAPI-04 and [^18^F]-FDG PET/CT for Primary Tumors

**Figure 1:**
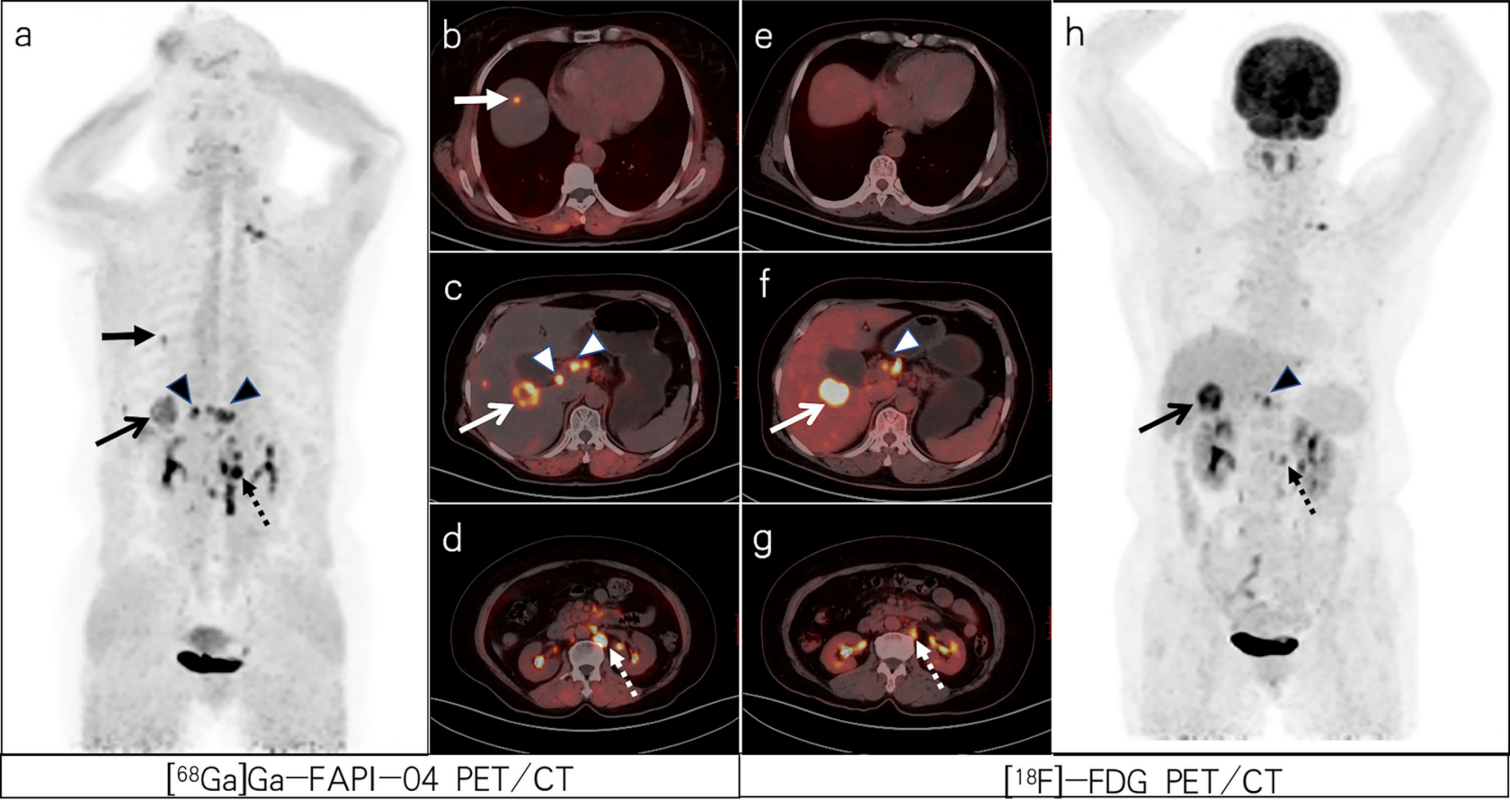
A 64-year-old woman with poorly differentiated gastric adenocarcinoma based on pathological biopsy under gastroscopy. Both [^68^Ga]Ga-FAPI-04 **(A, C)** and [^18^F]-FDG PET/CT **(F, H)** were negative in the primary focus. Compared with [^18^F]-FDG PET/CT **(E–G)**, [^68^Ga]Ga-FAPI-04 PET/CT **(B–D)** resulted in a higher liver/background ratio and identified more liver lesions (slender arrows, SUVmax=6.3 vs. 11.3) and para-aortic lymph node metastases (dashed arrows, SUVmax=12.8 vs. 8.0).

**Figure 2:**
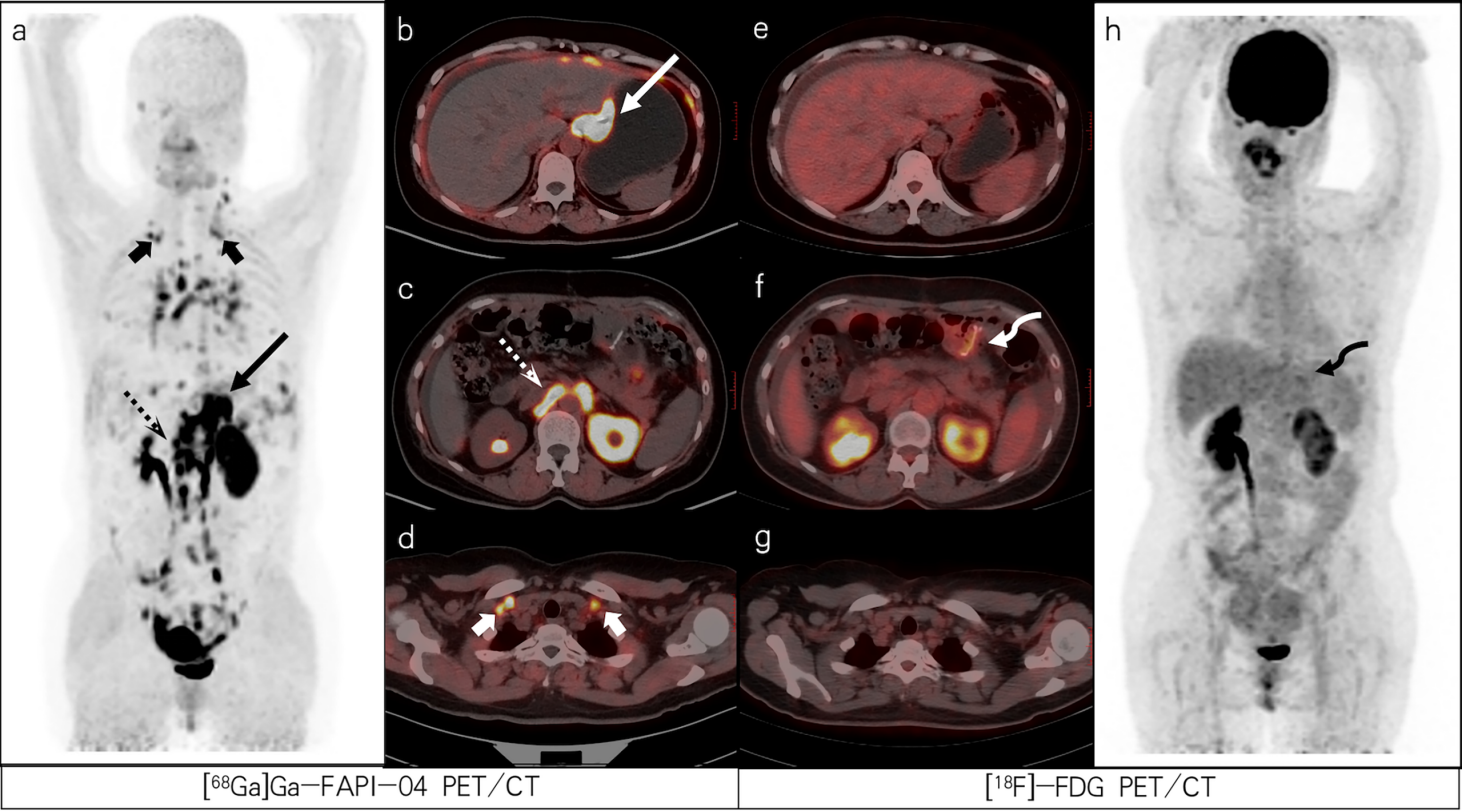
A 47-year-old female patient with poorly differentiated gastric adenocarcinoma with partial signet ring cells based on pathological biopsy after operation. [^68^Ga]Ga-FAPI-04 PET/CT **(A–D)** revealed high uptake in the gastric cardia (slender arrows, SUVmax=13.3), para-aortic lymph nodes (dashed arrows, SUVmax=15.7), and supraclavicular lymph nodes (short arrows, SUVmax=8.0) but negative uptake was observed on FDG PET/CT **(E–H)**. [^18^F]-FDG PET/CT **(F)** revealed increased uptake in the gastric anastomosis (bent arrow, SUVmax=3.2) but negative uptake was observed on [^68^Ga]Ga-FAPI-04 PET/CT **(C)**. The lesion was ultimately confirmed as residual gastritis by gastroscopic biopsy.

**Figure 3:**
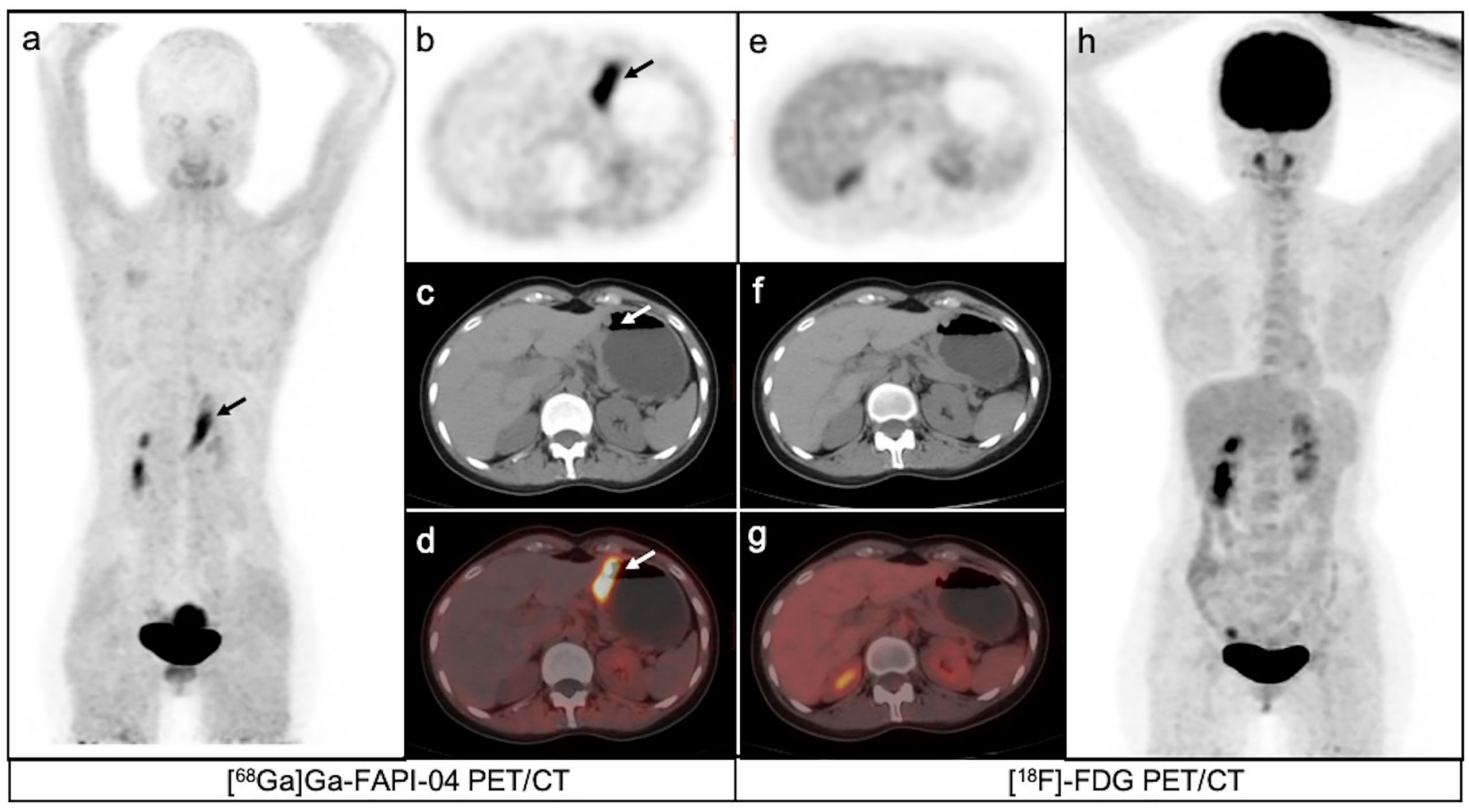
A 35-year-old female patient with moderately differentiated gastric adenocarcinoma based on pathological biopsy under gastroscopy. [^68^Ga]Ga-FAPI-04PET/CT **(A–D)** revealed high uptake in the primary tumor (arrows, SUVmax=8.3) but negative uptake was observed on [^18^F]-FDG PET/CT **(E–H)**.

**Figure 4:**
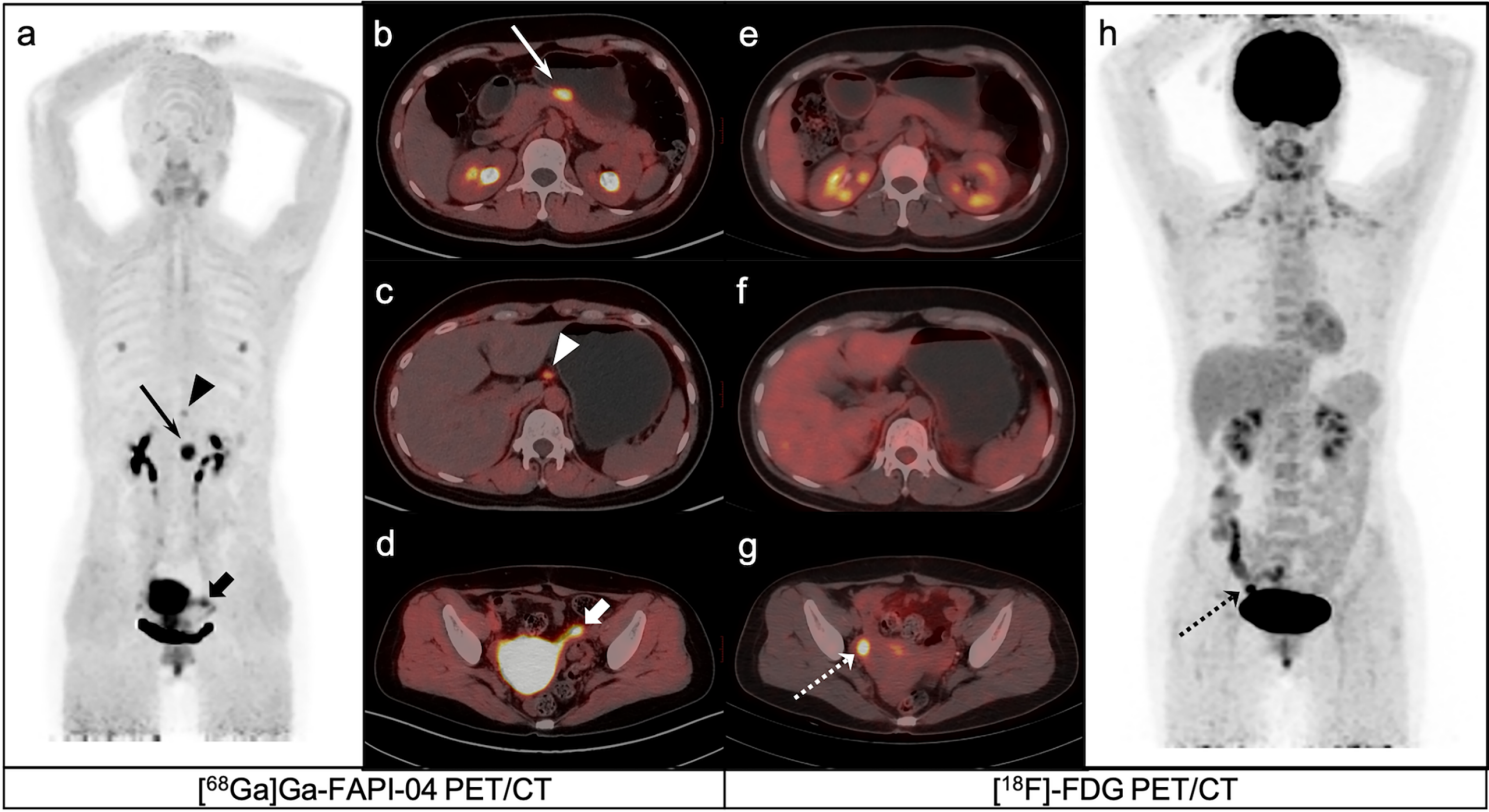
A 40-year-old female patient with poorly differentiated gastric adenocarcinoma based on pathological biopsy under gastroscopy. [^68^Ga]Ga-FAPI-04 PET/CT **(A–C)** revealed high uptake in the primary tumor (slender arrows, SUVmax=10.3) and lesser curved lymph nodes in the stomach (triangles, SUVmax=3.2), but negative uptake was observed on [^18^F]-FDG PET/CT **(E, F, H)**. [^18^F]-FDG PET/CT **(H)** revealed brown fat visualization at the base of the neck and in the supraclavicular fossa. [^18^F]-FDG PET/CT **(G)** revealed high uptake in the right ovarian (dashed arrows, SUVmax=8.2), but negative uptake was observed on [^68^Ga]Ga-FAPI-04 PET/CT **(D)**.

We identified 17 cases of newly diagnosed GC and 2 cases of postoperative primary recurrence of GC. Primary tumors were evaluated using [^68^Ga]Ga-FAPI-04 and [^18^F]-FDG PET/CT in 19 patients. [^68^Ga]Ga-FAPI-04 PET/CT exhibited a very high lesion detection rate (positive detection rate: 94.74% [18/19]) in primary gastric tumors, which was significantly higher than that of [^18^F]-FDG PET/CT (68.42% [13/19])(χ2 = 6.866, P < 0.01). The uptake of [^68^Ga]Ga-FAPI-04 in 19 cases of primary GC [SUVmax: 10.28(4.98,13.38)] was significantly higher than that of [^18^F]-FDG [SUVmax: 3.20(2.51,4.85)] (U=59.00, P < 0.01, **Table 2**). Primary lesions in one patient were not detected by either imaging agent (**Figure 1**), and primary lesions in four patients with newly diagnosed GC and one patient with recurrent GC were not detected by [^18^F]-FDG but were all detected by [^68^Ga]Ga-FAPI-04 PET/CT (**Figures 2**–**4**). One patient with GC with positive [^18^F]-FDG uptake but negative [^68^Ga]Ga-FAPI-04 uptake was confirmed by pathology to have remnant gastritis (**Figure 2**). The sensitivity, specificity, accuracy, positive predictive value (PPV), and negative predictive value (NPV) for primary lesion detection were 95% (19/20), 100% (5/5), 96% (24/25), 100% (19/19) and 83.3% (5/6) for [^68^Ga]Ga-FAPI-04; and 68.4% (13/19), 83.3% (5/6), 72% (18/25), 92.9% (13/14), and 45.4% (5/11) for [^18^F]-FDG, respectively.

**Table 2:** The SUVmax comparison between [^18^F]-FDG and [^68^Ga]Ga-FAPI-04 PET/CT in primary tumors, lymph node, and distant metastases.

Index	Primary tumors	Lymph node metastases	Distant metastases
FAPI SUVmax	10.28 (4.98,13.38)	9.20 (5.20,13.10)	8.00 (6.15,12.75)
FDG SUVmax	3.20 (2.51,4.85)	3.15 (1.50,7.20)	4.20 (2.05,9.12)
U	59.00	53.50	200.00
*p* value	< 0.01	< 0.01	< 0.01

### Diagnostic Efficiency of [^68^Ga]Ga-FAPI-04 and [^18^F]-FDG PET/CT for Lymph Node Metastasis

**Figure 5:**
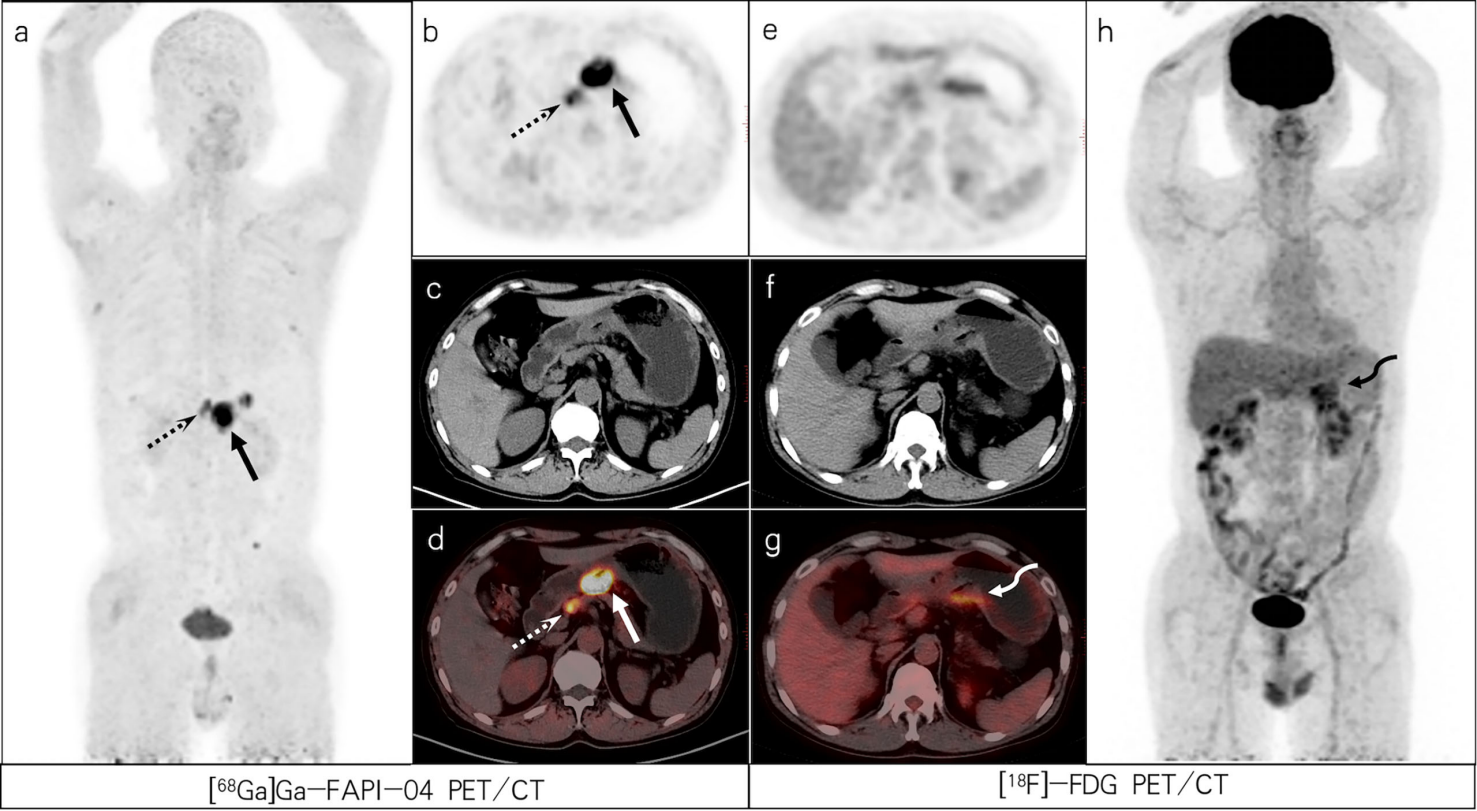
A 56-year-old male patient with poorly differentiated gastric adenocarcinoma based on pathological biopsy under gastroscopy. [^68^Ga]Ga-FAPI-04 PET/CT **(A–D)** revealed high uptake in the primary tumor (slender arrows, SUVmax=14.6) and lesser curved lymph nodes of the stomach (dashed arrows, SUVmax=8.7), but moderate uptake in the primary tumor (bent arrows, SUVmax=5.3) and negative uptake of lesser curved lymph nodes of the stomach were observed on [^18^F]-FDG PET/CT **(E–H)**.

Lymph nodes located around the gastric cavity were defined as area 1, perivascular lymph nodes as area 2, and perivisceral lymph nodes as area 3. In total, 75 positive lymph nodes in 12 of 25 patients were detected either by [^68^Ga]Ga-FAPI-04 or [^18^F]-FDG PET. Of 77 lymph nodes, 75 were correctly determined by [^68^Ga]Ga-FAPI-04 PET/CT(2 lymph nodes located in area 1 had false-positive uptake). In contrast, 32 out of 75 lymph nodes were correctly diagnosed by [^18^F]-FDG PET/CT (false-positive uptake in 4 lymph nodes located in area 1 and false-negative uptake in 43 lymph nodes). A total of 10 patients had area 1 lymph node metastasis, 3 had area 2 lymph node metastasis, and 3 had area 3 lymph node metastasis. One patient had lymph node metastasis in three regions, two patients had lymph node metastasis in two regions, and nine patients had only one regional lymph node metastasis. The 75 lymph node metastases comprised 53 lymph nodes in areas 1, 11 in areas 2, and 11 in area 3. Among the 75 nodal metastases, 43 (including 30 lymph nodes in area 1,10 lymph nodes in area 2, and 3 lymph nodes in area 3) were not detected by [^18^F]-FDG PET (**Figure 1**, **4** and **5**). The mean SUVmax of [^68^Ga]Ga-FAPI-04 in metastatic lymph nodes located in area 1 was higher than that of [^18^F]-FDG [8.90(4.43,10.65) vs. 3.15(1.28,11.4); U=30, P=0.130). For all metastatic lymph nodes, SUVmax values were significantly higher for [^68^Ga]Ga-FAPI-04 PET than for [^18^F]-FDG [9.20(5.20,13.10) vs. 3.15(1.50,7.20); U=53.50, P < 0.01) (**Table 2**). The sensitivity, specificity, accuracy, PPVand NPV for metastatic lymph nodes detection were 100% (75/75), 80% (8/10), 97.6% (83/85), 97.4% (75/77) and 100% (8/8) for [^68^Ga]Ga-FAPI-04; and 42.7% (32/75), 60% (6/10), 44.7% (38/85), 88.9% (32/36) and 12.2% (6/49) for [^18^F]-FDG, respectively. For the metastatic lymph nodes, there was no evidence of a higher lesion detection rate for [^68^Ga]Ga-FAPI-04 PET/CT than [^18^F]-FDG PET/CT(75/77 [97.40%] vs. 32/77 [41.56%], χ2 = 2.888, P =0.089).

### Diagnostic Efficiency of [^68^Ga]Ga-FAPI-04 and [^18^F]-FDG PET/CT for Distant Metastases

[^68^Ga]Ga-FAPI-04 PET/CT detected 275 distant metastases (18 supraclavicular lymph node, 22 mediastinal lymph node, 72 abdominal and pelvic lymph node, 2 lung, 4 liver, 5 bone, 20 bone marrow, 4 ovarian, and 128 peritoneum, omentum, mesentery metastases) in 12 patients. Based on the reference criteria, only 3 patients with distant metastasis in a single region were identified, including one case of ovarian metastasis, one case of supraclavicular fossa lymph node metastasis, and one case of abdominal lymph node metastasis. Six patients had extensive metastases (**Figure 1**, **2**). Distant metastases were localized in only the peritoneum, omentum, and mesentery in four patients; ovary in two patients; supraclavicular lymph nodes in one patient; ovary, peritoneum, omentum, mesentery, supraclavicular, mediastinal, and abdominal lymph nodes in one patient; liver, supraclavicular and abdominal lymph nodes in one patient; peritoneum, omentum, mesentery, mediastinal, and abdominal lymph nodes in one patient; peritoneum, omentum, mesentery, bone, and abdominal lymph nodes in one patient; lung, supraclavicular and mediastinal lymph nodes in one patient; ovary, peritoneum, omentum, mesentery, supraclavicular lymph nodes, and bone marrow in one patient; and abdominal lymph nodes in one patient. We identified one patient with lung, liver, and bone marrow metastasis; three patients with mediastinal lymph node metastasis; four patients with ovarian metastasis; five patients with cervical lymph node metastasis; and eight patients with peritoneum, omentum, and mesentery metastasis. [^18^F]-FDG PET/CT failed to detect 151 distant metastatic lesions in seven patients, including false-negative uptake in 80 peritoneum, omentum, and mesentery metastases; 36 abdominal lymph nodes metastases; 22 mediastinal lymph nodes metastases; 10 supraclavicular lymph nodes metastases (**Figure 2**); and 3 liver metastases (**Figure 1**). [^68^Ga]Ga-FAPI-04 PET/CT failed to detect eight distant metastatic lesions in two patients (one ovarian and seven bone metastases). The analysis of all distant metastases revealed that SUVmax value was significantly higher for [^68^Ga]Ga-FAPI-04 PET than for [^18^F]-FDG PET [8.00(6.15,12.75) vs. 4.20(2.05,9.12); U=200.00, P < 0.01) (**Table 2**). Based on all distant metastases analysis, [^68^Ga]Ga-FAPI-04 PET/CT showed higher lesion detection rate than [^18^F]-FDG PET/CT (275/283 [97.17%] vs. 122/283 [43.11%], χ2 = 11.858, P < 0.01).

### Changes in Tumor Staging and Oncological Management After [^68^Ga]Ga-FAPI-04 PET/CT

[^18^F]-FDG PET/CT was used to determine tumor staging in 10/17 newly diagnosed patients with GC and re-staging in 7/8 postoperative patients with GC. [^68^Ga]Ga-FAPI-04 PET/CT was used to determine tumor staging or restaging in 24 patients. In one patient, the primary tumor was not detected by [^68^Ga]Ga-FAPI-04, but all distant metastases were detected, indicating that [^68^Ga]Ga-FAPI-04 PET/CT still correctly defined staging (**Figure 1**). Ovarian metastasis in one patient was not detected by [^68^Ga]Ga-FAPI-04, which led to incorrect staging of the patient (**Figure 4**). [^68^Ga]Ga-FAPI-04 and [^18^F]-FDG PET/CT resulted in consistent staging or restaging in 17/25 patients. The perigastric lymph nodes of one newly diagnosed patient with GC and the primary tumor of one postoperative re-examined patient with GC were false-positive on [^18^F]-FDG imaging but negative on [^68^Ga]Ga-FAPI-04 imaging. [^68^Ga]Ga-FAPI-04 PET/CT correctly evaluated the staging and restaging of two patients (patients No. 7 and 23), thus avoiding overtreatment in these patients. Compared to [^18^F]-FDG PET/CT, [^68^Ga]Ga-FAPI-04 PET/CT revealed new positive lesions in 14/25 patients. For overall staging or restaging, 5/17 patients (patients No. 2, 11, 12, 13, and 14) with newly diagnosed GC were upstaged. [^18^F]-FDG PET/CT failed to detect primary tumors in four patients; perigastric lymph nodes in two patients; and peritoneum, omentum, and mesentery metastasis in one patient. However, [^68^Ga]Ga-FAPI-04 PET/CT provided the correct staging, thus enabling the patients to receive timely surgical treatment (patients No. 2, 11, and 13), chemotherapy, and targeted therapy (patients No. 12 and 14).(**Table 3**) The accuracy for tumor staging and restaging was 96% (24/25) for [^68^Ga]Ga-FAPI-04 and 72% (18/25) for [^18^F]-FDG, respectively.

**Table 3:** Changes in tumor staging and restaging by [^68^Ga]Ga-FAPI-04 PET/CT.

No.	Age(Y)	Sex	Tumor staging according to FDG	Tumor staging according to FAPI	Findings detected by FAPI but missed by FDG	Staging change
1	46	M	T3N1M1, IVB	T3N2M1, IVB	2 paracardial lymph nodes	None
2	35	F	Ne	T3N0M0, IIB	primary tumor	Up
3	53	F	T4aN2M1, IVB	T4aN2M1, IVB	None	None
4	67	F	T2N2M0, IIA	T2N2M0, IIA	None	None
5	40	F	T0N0M1, IVB	T3N2M0, III	2 paracardial lymph nodes, 1 lesser curved lymph node of stomach	Down
6	64	F	T0N2M1, IVB	T0N2M1, IVB	3 liver metastases, 1 left supraclavicular fossa lymph node, 20 abdominal lymph nodes	None
7	52	F	T1aN2M0, IIA	T1aN0M0, I	None	FDG(FP)
8	50	M	T1aN0M0, I	T1aN0M0, I	None	None
9	56	M	T4aN2M0, III	T4aN2M0, III	3 perigastric lymph nodes	None
10	70	F	T3N2M1, IVB	T3N2M1, IVB	None	None
11	43	M	Ne	T1N0M0, I	primary tumor	Up
12	79	M	T4aN0M0, IIB	T4aN0M1, IVB	peritoneal, omental, and mesenteric metastases>20	Up
13	56	M	Ne	T3N0M0, IIB	primary tumor	Up
14	68	M	Ne	T4aN1M1, IVB	primary tumor, 1 paracardial lymph node, 2 lesser curved lymph nodes of stomach, 9 omental and mesenteric metastases	Up
15	51	F	T1N0M1, IVB	T4aN2M1, IVB	6 perigastric lymph nodes, 3 abdominal lymph nodes, 20 omental and mesenteric metastases	None
16	53	M	T3N2M0, III	T3N2M0, III	None	None
17	37	F	T3N0M1, IVB	T3N0M1, IVB	4 omental and mesenteric metastases	None
18	70	M	TcN0M1, IVB	TcN0M1, IVB	None	None
19	40	F	TcN0M0	TcN0M0	None	None
20	66	F	TcN0M1, IVB	TcN0M1, IVB	None	None
21	58	M	TcN0M0	TcN0M0	None	None
22	54	M	TcN0M0	TcN0M0	None	None
23	73	M	T1aN0M0, I	TcN0M0	None	FDG(FP)
24	47	F	T0N0M1, IVB	T4aN3bM1, IVB	primary tumor, 6 perigastric lymph nodes, >20 distant lymph nodes, 20 omental and mesenteric metastases	None
25	66	M	T4aN0M1, IVB	T4aN1M1, IVB	2 para-celiac lymph nodes	None

Ne, negative; FP, false positive; Tc, the primary tumor was cut.

## Discussion

[^68^Ga]Ga-FAPI-04 is a potential imaging agent for tumor diagnosis that enables visualization of the stroma in the tumor microenvironment. The muscle and blood pool background of [^68^Ga]Ga-FAPI-04 is very low (SUVmax<2.0), resulting in a high tumor/normal tissue radioactivity ratio (tumor/non-tumor, T/NT) [8], which provides superior image quality compared to [^18^F]-FDG (10, 16, 17). Therefore, [^68^Ga]Ga-FAPI-04 PET/CT may have greater potential for application in tumors. In this study, we performed [^68^Ga]Ga-FAPI-04 and [^18^F]-FDG PET/CT in 25 patients with GC and compared the diagnostic efficiencies of the two imaging modalities for staging and restaging of GC. Compared to [^18^F]-FDG PET/CT, [^68^Ga]Ga-FAPI-04 PET/CT demonstrated a higher detection efficiency for primary tumors (18/19 [94.74%] vs. 13/19 [68.42%]), nodal metastases (75/77 [97.40%] vs. 32/77 [41.56%]), and distant metastases (275/283 [97.17%] vs. 122/283 [43.11%]) in patients with GC, which is similar to the results of Jiang et al. (18) and Kuten et al. (19). Compared to [^18^F]-FDG PET/CT, [^68^Ga]Ga-FAPI-04 PET/CT up-regulated tumor staging or restaging in 5/25 patients and down-regulated tumor staging or restaging in 2/25 patients. Accordingly, [^68^Ga]Ga-FAPI-04 PET/CT imaging is recommended for patients with GC who cannot be staged or re-staged clinically.

We observed that the detection rate of primary tumors of GC was significantly higher for [^68^Ga]Ga-FAPI-04 imaging than for [^18^F]-FDG imaging, and five patients were diagnosed using [^68^Ga]Ga-FAPI-04 but failed to be detected by [^18^F]-FDG. These patients included two cases of poorly differentiated adenocarcinoma, two cases of poorly differentiated adenocarcinoma with partial signet ring cell carcinoma, and one case of poorly differentiated adenocarcinoma with partial mucinous adenocarcinoma and signet ring cell carcinoma. The sensitivity of [^18^F]-FDG imaging for diagnosing signet ring cell carcinoma and mucinous adenocarcinoma is low (20, 21) because the expression levels of glucose transporter 1 (GLUT-1) in signet ring cell carcinoma and mucinous carcinoma are relatively low (22). Indeed, the physiological uptake of [^18^F]-FDG in the gastrointestinal tract is variable (23). In contrast, high FAP expression in matrix components of gastrointestinal tumors and metastatic tumors (9), rapid clearance by the kidney, less physiological uptake in normal organs, and high tumor-to-background ratio render [^68^Ga]Ga-FAPI-04 more advantageous for abdominal and pelvic imaging (5, 10, 16, 24). In this study, the SUVmax value of primary GC was significantly higher for [^68^Ga]Ga-FAPI-04 than for [^18^F]-FDG [10.28(4.98,13.38) vs. 3.20(2.51,4.85); U=59.00, P < 0.01]. Studies have reported that Claudin 18.2, inhibitors of the fibroblast growth receptor 2 pathway, and combinations of anti-angiogenic therapy and immune checkpoint blockade constitute novel targets for the treatment of GC (25–27). In this regard, [^68^Ga]Ga-FAPI-04 can be harnessed in PET imaging and may be a good molecular probe for radionuclide-targeted therapy.

In contrast to the results of Kuten et al. (19), the detection rate of primary tumors using [^68^Ga]Ga-FAPI-04 in this study did not reach 100%. In one case of poorly differentiated adenocarcinoma, the primary focus was a false negative in [^68^Ga]Ga-FAPI-04 and [^18^F]-FDG imaging, which may be related to the size and concealment of the lesion. No significant thickening of the gastric mucosa was observed on CT, but distant metastasis was detected using either imaging agent. However, the number of positive lesions on [^18^F]-FDG was significantly lower than that on [^68^Ga]Ga-FAPI-04 PET/CT.

Lymph node staging is key for the treatment and prognosis of patients with GC. Studies have demonstrated that PET/CT is useful for detecting occult lymph node metastases. The high detection rate of lymph nodes in [^68^Ga]Ga-FAPI-04 PET/CT contributes to the accurate staging of lymph nodes and may help to guide clinicians in developing appropriate treatment plans for GC. However, our study observed that inflammatory lymph nodes also took up [^68^Ga]Ga-FAPI-04, which is consistent with previous studies (14, 16). Inflammatory lymph nodes in one patient exhibited positive results for both imaging agents, indicating that [^68^Ga]Ga-FAPI-04 was not more specific than [^18^F]-FDG for detecting lymph node metastasis. However, [^68^Ga]Ga-FAPI-04 identified more postive lymph nodes compared to [^18^F]-FDG imaging (75 vs. 32).

Complete surgical resection of gastric tumors and adjacent lymph nodes with a negative cutting edge is currently the only effective treatment for GC. As resection is not possible for GC with distant metastasis, accurate staging of patients with GC is essential. Due to the limited number of pathological biopsies lesions, the negative predictive value of lesions may be incorrect, especially in distant metastases. Therefore, this study mainly discusses the positive distant metastatic lesions. In our study, peritoneum, omentum, mesentery metastasis was the most common distant metastasis (128/283 lesions). [^18^F]-FDG was observed in only 38 sites, whereas [^68^Ga]Ga-FAPI-04 detected all peritoneum, omentum, mesentery metastases. The low detection rate of [^18^F]-FDG for peritoneal metastasis is due to the relatively low expression of GLUT-1 in signet ring cell and mucinous carcinomas (22). When a tumor invades peritoneal tissues, a fibrotic response may occur, resulting in severe fibrosis. Therefore, [^68^Ga]Ga-FAPI-04 PET imaging may be more sensitive than [^18^F]-FDG for the diagnosis of GC peritoneal metastasis (5). In this study, one patient presented with liver metastasis, but only one liver metastasis with a size of 3.2 cm × 2.7 cm was detected by [^18^F]-FDG, while three additional smaller lesions were detected by [^68^Ga]Ga-FAPI-04, with the smallest lesion having a diameter of 0.6 cm. Our study demonstrated that [^68^Ga]Ga-FAPI-04 was superior to [^18^F]-FDG PET/CT for detecting liver metastasis, even small liver metastases with a diameter < 1 cm. Due to physiological [^18^F]-FDG uptake in the liver, [^18^F]-FDG PET/CT may not detect metastases with low [^18^F]-FDG uptake or small-sized metastases (6). Recent studies have demonstrated that physiological radioactivity uptake and liver background activity of [^68^Ga]Ga-FAPI-04 PET/CT in the gastrointestinal system are low (8, 11, 16), which is useful for detecting liver metastasis. The sensitivity of [^68^Ga]Ga-FAPI-04 for detecting liver metastasis in our study was 100%, which was similar to the findings of Ertan et al. (6). Only one patient in this study presented with liver metastasis, and a larger sample size is needed to verify our findings. In addition, the detection efficiency of [^68^Ga]Ga-FAPI-04 for distant lymph node metastasis was higher than that of [^18^F]-FDG, and the detection rates for supraclavicular fossa (18 vs. 8), mediastinum (22 vs. 0), and abdominal pelvic (72 vs. 36) lymph node metastases were significantly higher for [^68^Ga]Ga-FAPI-04 than for [^18^F]-FDG (P < 0.01). In this study, both imaging agents detected metastases of lung, accessory, bone, and bone marrow, but there was a higher uptake of imaging agents in the bone marrow, which may be due to the fact that patients with bone marrow metastasis are more likely to develop bone marrow fibrosis.

In one patient with bone metastasis, 12 bone metastases were detected by [^18^F]-FDG, while only five bone metastases were detected by [^68^Ga]Ga-FAPI-04. Accordingly, both imaging agents may be used for diagnosis in patients with bone metastasis of GC in order to avoid missed diagnosis. In addition, the ovarian metastasis of one patient was not detected by [^68^Ga]Ga-FAPI-04 PET/CT (**Figure 4**) due to physiological uptake by the uterus during [^68^Ga]Ga-FAPI-04 imaging, which may have caused false-negative uptake by the periuterine peritoneum, uterine rectal depression, and ovary. In this regard, enhanced MRI can be used to assist in diagnosis.

Our study had several limitations. First, the number of patients included in this study (n =25) was small, which precluded subgroup comparisons of GCs with different degrees of differentiation. We analyzed primary tumors, lymph nodes, and distant metastatic lesions of GC in a summarized way and compared tracer uptake and detection rates to obtain diagnostic efficiency. Second, owing to technical and ethical problems, it is impossible to biopsy all lymph nodes and distant metastases, and histopathological confirmation of all positive lesions cannot be used as a reference standard. Thus, the results of morphological and/or follow-up imaging were also used as the reference standard in our investigation.

In summary, [^68^Ga]Ga-FAPI-04 PET is superior to [^18^F]-FDG PET for the detection of primary tumors, lymph nodes, and distant metastases in patients with GC. Compared with [^18^F]-FDG PET/CT, [^68^Ga]Ga-FAPI-04 PET/CT corrected clinical stage in seven patients. In addition, the negative expression of [^68^Ga]Ga-FAPI-04 on PET/CT in patients with residual gastritis is helpful for distinguishing it from the high uptake of [^18^F]-FDG caused by inflammation. [^68^Ga]Ga-FAPI-04 has evident advantages for the detection of lymph node, peritoneal, omentum, mesentery and liver metastases. However, to confirm the definitive diagnostic value of [^68^Ga]Ga-FAPI-04 PET/CT for GC, our preliminary results warrant verification in other pathologically confirmed lesions.

## Data Availability Statement

The original contributions presented in the study are included in the article/Supplementary Material. Further inquiries can be directed to the corresponding authors.

## Ethics Statement

The studies involving human participants were reviewed and approved by Medical Research Ethics Committee, 2020035; Clinical Trials Registry No.ChiCTR2100044131. The patients/participants provided their written informed consent to participate in this study. Written informed consent was obtained from the individual(s) for the publication of any potentially identifiable images or data included in this article.

## Author Contributions

SZ, WW, TX, LQ and YC contributed to the study conception and design. Material preparation and data collection were performed by SZ, WW, LQ, YC, TX, HD, YL, HL, TD, YZ, LL. SZ, WW, YH processed and analysed the data. The first draft of the manuscript was written by SZ and LQ reviewed and revised the manuscript. All authors read and approved the final manuscript.

## Funding

This study was supported in part by research foundation projects from Luzhou Science & Technology Department (20107, 2021LZXNYD-J07), The Affiliated Hospital of Southwest Medical University (20087), and the nuclear medicine and molecular imaging key laboratory of Sichuan province open project (HYX18028).

## Conflict of Interest

The authors declare that the research was conducted in the absence of any commercial or financial relationships that could be construed as a potential conflict of interest.

## Publisher’s Note

All claims expressed in this article are solely those of the authors and do not necessarily represent those of their affiliated organizations, or those of the publisher, the editors and the reviewers. Any product that may be evaluated in this article, or claim that may be made by its manufacturer, is not guaranteed or endorsed by the publisher.

## References

[1] Smyth EC Nilsson M Grabsch HI van Grieken NCT Lordick F. Gastric Cancer. Lancet (2020) 396(10251):635–48. doi: 10.1016/s0140-6736(20)31288-5 32861308

[2] Horton KM Fishman EK. Current Role of Ct in Imaging of the Stomach. Radiographics A Rev Publ Radiol Soc North America Inc (2003) 23(1):75–87. doi: 10.1148/rg.231025071 12533643

[3] Young JJ Pahwa A Patel M Jude CM Nguyen M Deshmukh M. Ligaments and Lymphatic Pathways in Gastric Adenocarcinoma. Radiographics (2019) 39(3):668–89. doi: 10.1148/rg.2019180113 30951438

[4] Peng D He J Liu H Cao J Wang Y Chen Y. Fapi Pet/Ct Research Progress in Digestive System Tumors. Dig Liver Dis (2022) 54(2):164–9. doi: 10.1016/j.dld.2021.07.011 34364808

[5] Guo W Chen H. (68)Ga Fapi Pet/Ct Imaging in Peritoneal Carcinomatosis. Radiology (2020) 297(3):521. doi: 10.1148/radiol.2020202469 33048036

[6] Sahin E Elboga U Celen YZ Sever ON Cayirli YB Cimen U. Comparison of (68)Ga-Dota-Fapi and (18)Fdg Pet/Ct Imaging Modalities in the Detection of Liver Metastases in Patients With Gastrointestinal System Cancer. Eur J Radiol (2021) 142:109867. doi: 10.1016/j.ejrad.2021.109867 34315086

[7] Loktev A Lindner T Mier W Debus J Altmann A Jager D. A Tumor-Imaging Method Targeting Cancer-Associated Fibroblasts. J Nucl Med (2018) 59(9):1423–9. doi: 10.2967/jnumed.118.210435 PMC6126438 29626120

[8] Kratochwil C Flechsig P Lindner T Abderrahim L Altmann A Mier W. (68)Ga-Fapi Pet/Ct: Tracer Uptake in 28 Different Kinds of Cancer. J Nucl Med (2019) 60(6):801–5. doi: 10.2967/jnumed.119.227967 PMC6581228 30954939

[9] Kobayashi H Enomoto A Woods SL Burt AD Takahashi M Worthley DL. Cancer-Associated Fibroblasts in Gastrointestinal Cancer. Nat Rev Gastroenterol Hepatol (2019) 16(5):282–95. doi: 10.1038/s41575-019-0115-0 30778141

[10] Pang Y Zhao L Luo Z Hao B Wu H Lin Q. Comparison of (68)Ga-Fapi and (18)F-Fdg Uptake in Gastric, Duodenal, and Colorectal Cancers. Radiology (2021) 298(2):393–402. doi: 10.1148/radiol.2020203275 33258746

[11] Shi X Xing H Yang X Li F Li X. Comparison of Pet Imaging of Activated Fibroblasts and F-Fdg for Diagnosis of Primary Hepatic Tumors: A Prospective Pilot Study. Eur J Nucl Med Mol Imaging (2021) 48(5):1593–603. doi: 10.1007/s00259-020-05070-9 33097975

[12] Huang Y Cao J Peng D Liu H Chen Y. Bone Marrow Metastases From Gastric Adenocarcinoma on 68ga-Fapi-04 Pet/Ct. Clin Nucl Med (2022) 47(2):151–3. doi: 10.1097/RLU.0000000000003839 34319957

[13] Lin R Lin Z Zhang J Yao S Miao W. Increased 68ga-Fapi-04 Uptake in Schmorl Node in a Patient With Gastric Cancer. Clin Nucl Med (2021) 46(8):700–2. doi: 10.1097/RLU.0000000000003623 33826575

[14] Lan L Liu H Wang Y Deng J Peng D Feng Y. The Potential Utility of 68ga-Fapi-04 as a Novel Broad-Spectrum Tumor and Inflammatory Imaging Agent - Comparison With 18f-Fdg. Eur J Nucl Med Mol Imaging (2022) 49(3):963–79. doi: 10.1007/s00259-021-05522-w 34410435

[15] Frederik G Clemens K Thomas L Manfred M Anastasia L Wencke L. Fapi-Pet/Ct: Biodistribution and Preliminary Dosimetry Estimate of Two Dota-Containing Fap-Targeting Agents in Patients With Various Cancers. J Nucl Med (2019) 60(3):386–92. doi: 10.2967/jnumed.118.215913 PMC6424229 30072500

[16] Chen H Pang Y Wu J Zhao L Hao B Wu J. Comparison of [68ga]Ga-Dota-Fapi-04 and [18f] Fdg Pet/Ct for the Diagnosis of Primary and Metastatic Lesions in Patients With Various Types of Cancer. Eur J Nucl Med Mol Imaging (2020) 47(8):1820–32. doi: 10.1007/s00259-020-04769-z 32222810

[17] Wang S Zhou X Xu X Ding J Liu S Hou X. Clinical Translational Evaluation of Al18f-Nota-Fapi for Fibroblast Activation Protein-Targeted Tumor Imaging. Eur J Nucl Med Mol Imaging (2021) 48(13):4259–71. doi: 10.1007/s00259-021-05470-5 34165601

[18] Jiang D Chen X You Z Wang H Zhang X Li X. Comparison of [(68) Ga]Ga-Fapi-04 and [(18)F]-Fdg for the Detection of Primary and Metastatic Lesions in Patients With Gastric Cancer: A Bicentric Retrospective Study. Eur J Nucl Med Mol Imaging (2022) 49(2):732–42. doi: 10.1007/s00259-021-05441-w 34297193

[19] Kuten J Levine C Shamni O Pelles S Wolf I Lahat G. Head-To-Head Comparison of [(68)Ga]Ga-Fapi-04 and [(18)F]-Fdg Pet/Ct in Evaluating the Extent of Disease in Gastric Adenocarcinoma. Eur J Nucl Med Mol Imaging (2022) 49(2):743–50. doi: 10.1007/s00259-021-05494-x PMC8803763 34302504

[20] Zhu A Lee D Shim H. Metabolic Pet Imaging in Cancer Detection and Therapy Response. Semin Oncol (2011) 38(1):55–69. doi: 10.1053/j.seminoncol.2010.11.012 21362516 PMC3075495

[21] Turlakow A Yeung HW Salmon AS Macapinlac HA Larson SM. Peritoneal Carcinomatosis: Role of (18)F-Fdg Pet. J Nucl Med (2003) 44(9):1407–12. doi: 10.1637/7955-022307-Reg 12960184

[22] Kawamura T Kusakabe T Sugino T Watanabe K Fukuda T Nashimoto A. Expression of Glucose Transporter-1 in Human Gastric Carcinoma : Association With Tumor Aggressiveness, Metastasis, and Patient Survival. Cancer (2001) 92(3):634–41. doi: 10.1002/1097-0142(20010801)92:3<634::aid-cncr1364>3.0.co;2-x 11505409

[23] Akin EA Qazi ZN Osman M Zeman RK. Clinical Impact of Fdg Pet/Ct in Alimentary Tract Malignancies: An Updated Review. Abdom Radiol (2020) 45(4):1018–35. doi: 10.1007/s00261-020-02447-0 32152644

[24] Frederik G Clemens K Thomas L Manfred M Anastasia L Wencke L. Fapi-Pet/Ct: Biodistribution and Preliminary Dosimetry Estimate of Two Dota-Containing Fap-Targeting Agents in Patients With Various Cancers. J Nucl Med (2019)60(3):386–392. doi: 10.2967/jnumed.118.215913 30072500 PMC6424229

[25] Cancer Genome Atlas Research Network. Comprehensive Molecular Characterization of Gastric Adenocarcinoma. Nature (2014) 513(7517):202–9. doi: 10.1038/nature13480 PMC4170219 25079317

[26] Tejani MA Cheung E Eisenberg PD Scott AJ Catenacci D. Phase I Results From the Phase 1/3 Fight Study Evaluating Bemarituzumab and Mfolfox6 in Advanced Gastric/Gej Cancer (Gc). J Clin Oncol (2019) 37(4_suppl):91–1. doi: 10.1200/JCO.2019.37.4_suppl.91

[27] Takahari D Shoji H Hara H Esaki T Kato K. (2018). Preliminary Result of Phase 1/2 Study of Ramucirumab Plus Nivolumab in Patients With Previously Treated Advanced Gastric Adenocarcinoma (NivoRam study). J Clin Oncol (2018) 6(1). doi: 10.1200/JCO.2018.36.15_suppl.4047

